# Improving HER2 testing reproducibility in HER2-low breast cancer

**DOI:** 10.20517/cdr.2022.29

**Published:** 2022-09-01

**Authors:** Elham Sajjadi, Konstantinos Venetis, Mariia Ivanova, Nicola Fusco

**Affiliations:** ^1^Division of Pathology, IEO, European Institute of Oncology IRCCS, Milan 20141, Italy.; ^2^Department of Oncology and Hemato-Oncology, University of Milan, Milan 20122, Italy.

**Keywords:** Breast cancer, biomarkers, HER2, HER2 low, targeted therapy, antibody-drug conjugates, immunohistochemistry, ISH

## Abstract

HER2 is a pillar biomarker in breast cancer, and it is assessed by immunohistochemistry (IHC) using a three-tier scoring system and reflex *in situ* hybridization (ISH) for IHC score 2+. Novel HER2-directed antibody-drug conjugates have demonstrated significant antitumor activity in breast cancers with low levels of HER2 expression, i.e. IHC score 1+ or ISH-negative IHC score 2+. Both primary and acquired resistance to anti-HER2 therapies remains a challenge in the treatment of breast cancers according to the HER2 positivity *continuum*. Thus, the ability to precisely discriminate among HER2-zero, HER2-low, and HER2-positive breast cancers is no longer a mere academic exercise. HER2 testing criteria, guidelines, and quality controls are re-gaining momentum for this new clinical need. Therefore, all preanalytical and analytical variables that might trouble the sensitivity and reproducibility of this test should be carefully considered to address all possible issues and open all possible therapeutic opportunities for breast cancer patients.

## INTRODUCTION: THE EXPANDED SPECTRUM OF HER2 POSITIVITY IN BREAST CANCER

HER2 status assessment is considered “the” predictive test in breast cancer pathology because of its extremely relevant prognostic and predictive values^[1]^. Testing for HER2 consists of immunohistochemistry (IHC) using a three-tier scoring system and *in situ* hybridization (ISH) in case of IHC score 2+^[2]^. Using this method, patients with HER2+ breast cancer, i.e. IHC score 3+ or ISH-positive IHC score 2+ are eligible for anti-HER2 targeted therapy^[3,4]^. Recently, the DESTINY-Breast04 clinical trial demonstrated that targeting HER2 provides significant benefits also for patients with metastatic breast cancer showing low levels of HER2 expression, i.e. IHC score 1+ or ISH-negative IHC score 2+^[5]^. Of note, patients with hormone receptor (HR)+ disease had to be refractory/resistant to endocrine therapy (ET). In this randomized clinical study, the antibody-drug conjugate (ADC) trastuzumab deruxtecan (T-DXd) improved median progression-free survival by 4.8 months and median overall survival by 6.6 months, compared with standard single-agent chemotherapy. These data establish a new standard of care for patients with HER2-low breast cancer and a fast-track approval for T-DXd is expected. Indeed, these tumors were previously not eligible for HER2 targeting because they were considered resistant to HER2 inhibitor monoclonal antibodies^[6]^. Due to these groundbreaking advances, the capability to precisely discriminate among HER2-zero, HER2-low, and HER2+ breast cancers is no longer a mere academic exercise for pathologists. Instead, HER2 testing criteria, guidelines, and quality controls are re-gaining momentum to embrace this new upcoming clinical need.

## THE EVOLUTION OF HER2 TESTING

### Historical perspective

The American Society of Clinical Oncology (ASCO)/College of American Pathologists (CAP) Expert Panel has made remarkable efforts to improve the analytical reliability of HER2 testing. Since 2007, they have provided guidelines on HER2 interpretation in breast cancer, including algorithms for defining positive, equivocal, and negative values for both HER2 protein expression and gene amplification. Specifically, a positive HER2 result was IHC staining of 3+ (i.e., uniform, intense membrane staining of > 30% of invasive tumor cells), a fluorescent ISH (FISH) result > 6 HER2 gene copies per nucleus, or a HER2 gene to chromosome 17 signals ratio > 2.2; a negative result was an IHC staining of 0 or 1+, a FISH result of < 4.0 HER2 gene copies per nucleus, or FISH ratio < 1.8^[7]^. Subsequently, in 2009, the intra-tumor heterogeneity of HER2 amplification was supplemented to the guideline and defined as HER2/CEP17 signal ratios > 2.2 in 5%-50% of the neoplastic cells^[8,9]^. Later, in 2013, to avoid false-negative results, the positivity threshold was decreased to 10% and the HER2/CEP17 ratio to 2^[10]^. With these updates, a considerably higher number of patients could be treated with trastuzumab with a reasonable, slight increase of adverse events. In 2018, ASCO/CAP released an integration to the 2013 edition, emphasized the coordination between IHC and ISH results, and mostly addressed specific clinical questions and technical issues related to HER2 equivocal and heterogeneity^[3]^. The current IHC algorithm with the expanded spectrum of HER2 reporting category and the reporting results for ISH assays are shown in Tables 1 and 2, respectively (Source: Breast Biomarker Reporting, CAP Cancer Protocol Templates, v1.4.1.1, November 2021 update, available at: https://documents.cap.org/protocols/Breast.Bmk_1.4.1.1.REL_CAPCP.pdf.

**Table 1:** Reporting results of HER2 testing by immunohistochemistry and the corresponding expanded spectrum of positivity (ASCO/CAP 2021)

**Score**	**Membrane staining pattern**	**Tumor cells**	**Classical category**	**Expanded spectrum**
3+	Intense, complete (circumferential)	> 10%	HER2+	HER2+
2+	Weak-to-moderate, complete (circumferential)	> 10%	HER2+ if ISH+ and HER2- if ISH-	HER2+ if ISH+ and HER2-low if ISH-
	Intense, complete	≤ 10%		
1+	Faint/barely perceptible, incomplete	> 10%	HER2-	HER2-low
0	Faint/barely perceptible, incomplete	≤ 10%	HER2-	HER2 ultra low
	No staining		HER2-	HER2-zero

IHC: Immunohistochemistry; HER2: human epidermal growth factor receptor 2.

**Table 2:** Reporting results of HER2 testing by in situ hybridization (ASCO/CAP 2021)

Result	Assay	Criteria
Positive	Single probe	CN ≥ 6.0 CN ≥ 4.0 and CN < 6.0 and IHC = 3+ CN ≥ 4.0 and CN < 6.0 and dual ISH ratio ≥ 2.0
	Dual probe	Ratio ≥ 2.0 and CN ≥ 4.0 Ratio ≥ 2.0 and CN < 4.0 and IHC = 3+ Ratio < 2.0 and CN ≥ 6.0 and IHC > 1+ Ratio < 2.0 and 4.0 ≤ CN > 6.0 and IHC = 3+
Negative	Single probe	CN < 4.0 CN ≥ 4.0 and CN < 6.0 and IHC < 3+ CN ≥ 4.0 and CN < 6.0 and dual ISH ratio < 2.0 and CN < 4.0
	Dual probe	Ratio ≥ 2.0 and CN < 4.0 and IHC < 3+ Ratio < 2.0 and CN ≥ 6.0 and IHC < 2+ Ratio < 2.0 and 4.0 ≤ CN > 6.0 and IHC < 3+ Ratio < 2.0 and CN < 4.0

CN: Average HER2 copy number (signals/cell); IHC: immunohistochemical score; ratio: HER2/CEP17 ratio.

The evolution of different ISH types has established a proposal of reliable FISH alternatives with faster performance at a lower cost. Silver *in situ* hybridization and chromogenic *in situ* hybridization have demonstrated great reproducibility and have been approved by the U.S. Food and Drug Administration for HER2 testing in breast cancer. The combined technology of dual-color dual-hapten *in situ* hybridization (D-DISH), providing the convenience of light microscopy with an ISH specificity, using chromogenic probes instead of fluorescent ones, also demonstrates a high reproducibility^[11]^. Gene protein assays and next-generation sequencing are the newer modalities, which may potentially help to overcome the issue of the tumor heterogeneity, combining the benefits of both IHC and ISH by single-slide HER2 status assessment^[12]^. Other promising candidates for the alternative HER2 status assessment are molecular (multiplex ligation-dependent probe amplification) and liquid biopsy assays using circulating tumor cells, cell-free tumor DNA (ctDNA), and extracellular vesicles, which have been tested in IHC/ISH HER2-equivocal breast cancers. Transcriptomic analysis has revealed high *HER2* mRNA levels in HER2-enriched breast cancer subtypes using the PAM50 probe, suggesting another possible subtype signature. Dual HER2-blockade led to positive results in patients with higher *HER2* mRNA levels; thus, it is a newly proposed biomarker for chemotherapy de-escalation^[13-15]^. Despite evolving modalities of HER2 testing, they are currently awaiting approval and the IHC-ISH approach remains the “gold standard”^[16]^.

### HER2 targeting and biomarkers of drug resistance in breast cancer

The canonical HER2+ breast cancers account for 15%-20% of all breast malignancies and are characterized by the overexpression/amplification of HER2^[9]^. These patients can be treated with a variety of anti-HER2 compounds, including trastuzumab and pertuzumab, in both adjuvant and neoadjuvant settings^[17,18]^. Regrettably, the development of primary or acquired resistance is not uncommon in these patients^[19]^. Furthermore, despite the success of ET-combined therapy in the metastatic setting, a substantial proportion of initial responders eventually developing resistance and/or recurrence over the time. In this respect, novel predictive biomarkers are warranted. Several mechanisms have been proposed thus far, including phenotype shift through selective pressure^[20-23]^. Interestingly, it has been observed that low levels of HER2 expression, both in terms of the percentage of HER2-expressing cells and IHC staining intensity, may impair the binding of traditional anti-HER2 agents to the target^[24]^. An additional mechanism of resistance is represented by the activation of the phosphatidylinositol-3-kinase (PI3K)/Akt and the mammalian target of rapamycin (PI3K/AKT/mTOR) and cyclin D1-dependent kinase 4/6 (CDK4/6) pathways^[25-27]^. Finally, genetic alterations in HER2 regions responsible for kinase activation can compromise the ability of anti-HER2 compounds to bind HER2. Specifically, studies have shown that mutations in positions L755S and T798I can cause the highest levels of resistance^[28,29]^. In this scenario, an appropriate and reproducible HER2 test able to precisely identify the various types of HER2 expression is essential to put in place the most appropriate HER2-targeted treatment combination.

### Challenges and barriers for HER2-low identification

The current evidence on HER2-targeting therapies in HER2-low breast cancer arises from several translational research studies employing various classes of monoclonal antibodies, anti-HER2 vaccines, cellular immunotherapy, ADC, and bispecific antibodies^[22]^. In particular, ADC combines the selectivity of anti-HER2-targeted therapy with the cytotoxic potency of chemotherapy while reducing the systemic exposure to the cytotoxic payload^[30-32]^. Among this class of drugs, T-DXd is composed of a humanized anti-human HER2 antibody, an enzymatically cleavable peptide-linker, and a proprietary topoisomerase I inhibitor^[30]^. After the cleavage of the peptide-based linker by lysosomal cathepsins, the released cytotoxic molecules can affect HER2-low cells neighboring those with higher HER2 expression, reflecting a bystander effect; thus, T-DXd has shown the ability to target tumor cells regardless of their HER2-status^[33,34]^. These findings have transformed the traditional dichotomy of HER2 status and have raised the expectations in this field. However, whether HER2-low breast cancer represents a distinct biologic and prognostic subtype is still a matter of controversy. Recently, an analysis of 5,235 patients with HER2-negative invasive breast cancer revealed that HER2-low expression was positively associated with level of ER expression, and ER-low tumors were enriched among HER2-zero tumors^[35]^. The Authors of this study suggest that the worse prognosis of ER-low tumors might confound the prognostic analyses of HER2-low expression. Perhaps, re-testing and re-assess HER2 status in these retrospective studies, particularly for HER2-zero tumors would provide additional information. Assessment of data from CAP surveys and from a Yale University-based study of concordance of 18 pathologists reading 170 breast cancer biopsies revealed that the scoring accuracy for HER2 IHC in the low range (0 *vs*. 1+) was poor^[36]^. This inaccuracy might lead to misassignment of many patients for treatment with T-DXd. Further development of HER2-low breast cancer treatment includes combined treatment strategies evaluation, where high expectations rely on the ADC and immune checkpoint inhibitor combination^[37]^.

### Reducing false-negative and false-“low-positive” results

It has been reported that up to 55% of patients with breast cancer have a HER2-low disease^[38-41]^. However, up to now, discriminating between IHC score 0 with incomplete and faint staining in ≤ 10% of tumor cells and score 1+, as well as between score 1+ with faint staining and score 2+ with weak-to-moderate staining intensity, was a negligible pathology exercise^[42,43]^. In this context, updated guidelines for all the analytical phases are necessary to improve our diagnostic sensitivity for HER2-low breast cancer^[43]^. Although the ASCO/CAP continuously updates the guidelines to ensure optimal diagnostic performance, the interpretation of HER2 IHC in HER2-low breast cancer has not yet been formally endorsed^[44]^. Accurate and reproducible testing strategies and techniques are the key aspects to identifying patients who may benefit from ADC^[43]^. In this regard, to prevent false-negative/positive results and subsequent mistreatment, careful supervision of preanalytical and analytical issues is required^[44]^. A range of preanalytical factors such as fixation, antigen retrieval, antibody clones, enzymatic activity, reaction time, temperature, and substrate concentration may influence HER2 staining intensity^[45,46]^. Staining methodology, particularly antigen retrieval, the availability of diverse antibody clones with various specificity [i.e., PATHWAY® HER2 (clone 4B5; Ventana Medical Systems Inc., Tucson, AZ, USA), HercepTest™ (Dako Denmark A/S, Glostrup, Denmark), and Oracle® HER2 (clone CB11; Leica Microsystems GmbH, Wetzlar, Germany)] may significantly impact the accuracy and reproducibility of results and complicate the identification of HER2-low expression in terms of false-positive and false-negative results^[46,47]^. Test repetition for equivocal results may exclude possible technical problems; however, it does not often result in definitive positive or negative results. Regarding the post-analytical phase, the lack of consistent epithelial internal positive control for HER2 within non-neoplastic breast tissue, HER2 intratumoral heterogeneity, particularly in HER2-low breast cancer, and the semi-quantitative and subjective mode of HER2 assessment may lead to inter-observer variability affecting the accuracy of the results^[44,48,49]^. A larger number of patients with HER2+ metastatic breast cancer might possibly have a long-lasting clinical response with the careful application of mor precise and reproducible diagnostic methods^[50]^. Thus, rigorous quality control procedures for specimen preparation coupled with well-defined guidelines for the precise assessment of HER2-low cases are required^[51,52]^.

## CONCLUSION

In light of the therapeutic armamentarium broadening based on HER2 patterns of expression, HER2 testing is becoming more complex. To improve sensitivity, specificity, and reproducibility of HER2 status evaluation, pathologists need to be aware of the molecular mechanisms underlying the variable expression of HER2 in different types of breast cancers and even in different cells within the same tumor. In this respect, continuous education, update, quality controls, and multidisciplinary discussions are key elements for optimal patient management.

## DECLARATIONS

### Copyright

© The Author(s) 2022.
